# Hyperparameter Optimizer with Deep Learning-Based Decision-Support Systems for Histopathological Breast Cancer Diagnosis

**DOI:** 10.3390/cancers15030885

**Published:** 2023-01-31

**Authors:** Marwa Obayya, Mashael S. Maashi, Nadhem Nemri, Heba Mohsen, Abdelwahed Motwakel, Azza Elneil Osman, Amani A. Alneil, Mohamed Ibrahim Alsaid

**Affiliations:** 1 Department of Biomedical Engineering, College of Engineering, Princess Nourah bint Abdulrahman University, P.O. Box 84428, Riyadh 11671, Saudi Arabia; 2 Department of Software Engineering, College of Computer and Information Science, King Saud University, Riyadh 11543, Saudi Arabia; 3 Department of Information Systems, College of Science & Art at Mahayil, King Khalid University, Abha 62529, Saudi Arabia; 4 Department of Computer Science, Faculty of Computers and Information Technology, Future University in Egypt, New Cairo 11835, Egypt; 5 Department of Information Systems, College of Business Administration in Hawtat Bani Tamim, Prince Sattam Bin Abdulaziz University, Al-Kharj 16278, Saudi Arabia; 6 Department of Computer and Self Development, Preparatory Year Deanship, Prince Sattam Bin Abdulaziz University, Al-Kharj 16278, Saudi Arabia

**Keywords:** decision making, healthcare, breast cancer classification, histopathological images, deep learning

## Abstract

**Simple Summary:**

This study develops an arithmetic optimization algorithm with deep-learning-based histopathological breast cancer classification (AOADL-HBCC) technique for healthcare decision making. The AOADL-HBCC technique employs noise removal based on median filtering (MF) and a contrast enhancement process. In addition, the presented AOADL-HBCC technique applies an AOA with a SqueezeNet model to derive feature vectors. Finally, a deep belief network (DBN) classifier with an Adamax hyperparameter optimizer is applied for the breast cancer classification process.

**Abstract:**

Histopathological images are commonly used imaging modalities for breast cancer. As manual analysis of histopathological images is difficult, automated tools utilizing artificial intelligence (AI) and deep learning (DL) methods should be modelled. The recent advancements in DL approaches will be helpful in establishing maximal image classification performance in numerous application zones. This study develops an arithmetic optimization algorithm with deep-learning-based histopathological breast cancer classification (AOADL-HBCC) technique for healthcare decision making. The AOADL-HBCC technique employs noise removal based on median filtering (MF) and a contrast enhancement process. In addition, the presented AOADL-HBCC technique applies an AOA with a SqueezeNet model to derive feature vectors. Finally, a deep belief network (DBN) classifier with an Adamax hyperparameter optimizer is applied for the breast cancer classification process. In order to exhibit the enhanced breast cancer classification results of the AOADL-HBCC methodology, this comparative study states that the AOADL-HBCC technique displays better performance than other recent methodologies, with a maximum accuracy of 96.77%.

## 1. Introduction

Cancer is one of the most serious health concerns that threaten the health and lives of individuals [1]. The mortality rate and incidence of breast cancer seem to be increasing in recent times. Early precise diagnosis is considered to be a key to enhancing the chances of survival. The primary step in initial diagnosis is a mammogram, but it can be difficult to identify tumors in dense breast tissue, and X-ray radiation imposes a risk to the radiologist’s and the patient’s health [2]. The precise diagnosis of breast cancer requires skilled histopathologists, as well as large amounts of effort and time for task completion. Furthermore, the diagnosis outcomes of various histopathologists are not the same, because they mainly depend on the former knowledge of each histopathologist [3]. The average diagnosis precision is just 75%, which leads to low consistency in diagnoses. The term histopathology can be defined as the process of detailed evaluation and microscopic inspection of biopsy samples carried out by a pathologist or expert to learn about cancer growth in tissues or organs [4]. Common histopathological specimens have more structures and cells that can be dispersed and surrounded haphazardly by distinct types of tissues [5]. The physical analysis of historic pictures, along with the visual observation of such images, consumes time. This necessitates expertise and experience. In order to raise the predictive and analytical capabilities of histopathological images, the utility of computer-based image analysis represents an effective method [6]. This form of analysis is even efficient for histopathological images because it renders a dependable second opinion for consistent study, which increases output. This could aid in curtailing the time it takes to identify an issue. Thus, the burden on pathologists and the death rate can be minimized [7].

Today, machine learning (ML) is fruitfully enforced in text classification, image recognition, and object recognition. With the progression of computer-aided diagnosis (CAD) technology, ML is effectively implemented in breast cancer diagnosis [8]. Histopathological image classification related to conventional ML techniques and artificial feature extraction demands a manual model of features; however, it does not need an apparatus with more efficiency, and it has benefits in the computing period [9]. However, histopathological image classification related to deep learning (DL), particularly convolutional neural networks (CNNs), frequently needs a large number of labelled training models, whereas the labelled data are hard to gain [10]. The labeling of lesions is laborious and time-consuming work, even for professional histopathologists.

This study develops an arithmetic optimization algorithm with deep-learning-based histopathological breast cancer classification (AOADL-HBCC) technique for healthcare decision making. The presented AOADL-HBCC technique mainly aims to recognize the presence of breast cancer in HIs. At the primary level, the AOADL-HBCC technique employs noise removal based on median filtering (MF) and a contrast enhancement process. In addition, the presented AOADL-HBCC technique applies an AOA with a SqueezeNet model to derive feature vectors. Finally, a deep belief network (DBN) classifier with an Adamax hyperparameter optimizer is applied for the breast cancer classification process. In order to exhibit the enhanced breast cancer classification results of the AOADL-HBCC approach, a wide range of simulations was performed. 

## 2. Related Works

Shankar et al. [11] established a new chaotic sparrow search algorithm including a deep TL-assisted BC classification (CSSADTL-BCC) technique on histopathological images (HPIs). The projected technique mostly concentrated on the classification and detection of BC. To realize this, the CSSADTL-BCC system initially carried out a Gaussian filter (GF) system for eradicating the presence of noise. In addition, a MixNet-oriented extracting feature system was utilized for generating a suitable group of feature vectors. Furthermore, a stacked GRU (SGRU) classifier system was utilized for allotting classes. In [12], TL and deep extracting feature approaches were employed that adjusted a pretraining CNN system to the current problem. The VGG16 and AlexNet methods were considered in the projected work for extracting features and AlexNet was employed for additional finetuning. The achieved features were then classified by SVM. 

Khan et al. [13] examined a new DL infrastructure for the classification and recognition of BC from breast cytology images utilizing the model of TL. Generally, DL infrastructures demonstrated that certain problems were accomplished in isolation. In the presented structure, features in images were extracted employing pretrained CNN infrastructures such as ResNet, GoogLeNet, and VGGNet that are provided as fully connected (FC) layers to classify benign and malignant cells employing an average pooling classifier. In [14], a DL-related TL system was presented for classifying histopathological images automatically. Two famous and present pretrained CNN techniques, DenseNet161 and ResNet50, were trained as well as tested via grayscale and color images.

Singh et al. [15] examined a structure dependent upon the concept of TL for addressing this problem and concentrated their efforts on HPI and imbalanced image classifiers. The authors utilized common VGG19 as the base method and complemented it with different recent approaches for improving the entire efficiency of the technique. In [16], the conventional softmax and SVM-classifier-related TL systems were estimated for classifying histopathological cancer images in a binary BC database and a multiclass lung and colon cancer database. For achieving optimum classifier accuracy, a procedure that assigns an SVM technique to an FC layer of softmax-related TL techniques was presented. In [17], the authors’ concentration on BC in HPI was attained by utilizing microscopic scans of breast tissues. The authors proposed two integrated DCNNs for extracting well-known image features utilizing TL. The pretrained Xception and Inception techniques were utilized in parallel. Afterwards, feature maps were integrated and decreased by dropout before they provided the final FC layer to classify.

## 3. The Proposed Model

**Figure 1:**
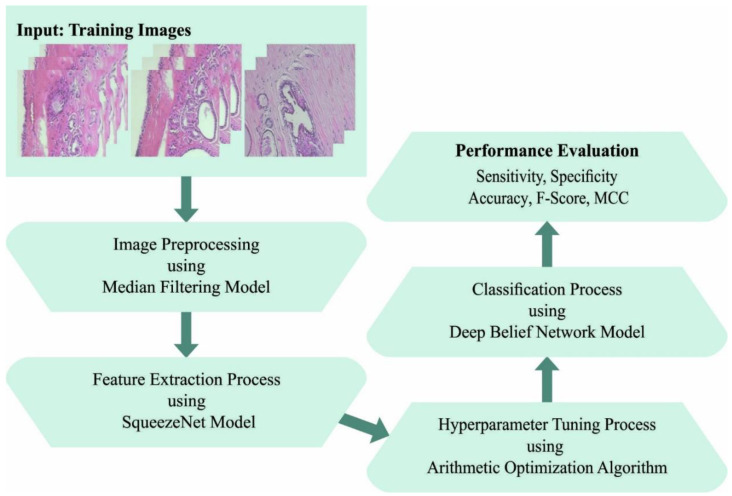
Block diagram of AOADL-HBCC system.

In this work, an automated breast cancer classification method, named the AOADL-HBCC technique, was developed using HIs. The presented AOADL-HBCC technique mainly aims to recognize the presence of breast cancer in HIs. It encompasses a series of processes, namely SqueezeNet feature extraction, AOA hyperparameter tuning, DBN classification, and an Adamax optimizer. Figure 1 shows a block diagram of the AOADL-HBCC mechanism.

### 3.1. Design of AOA with SqueezeNet Model

**Figure 2:**
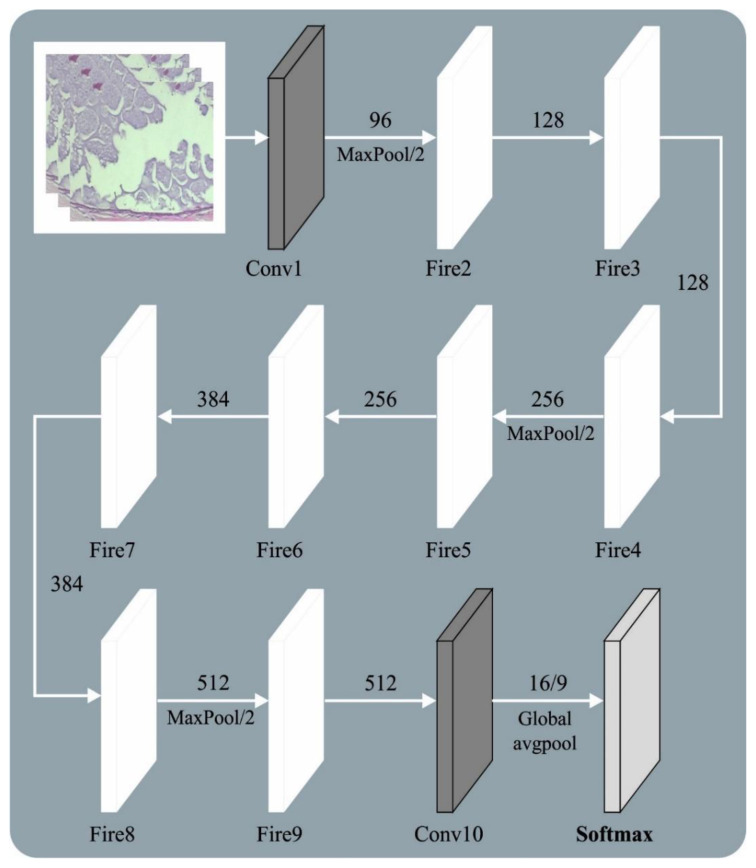
Architecture of SqueezeNet model.

In this study, the presented AOADL-HBCC technique utilized an AOA with a SqueezeNet model to derive feature vectors. Presently, GoogLeNet, ResNet, VGG, AlexNet, etc., are signature techniques of DNN [18]. However, deep networks might lead to remarkable performance; this method is trained and recognition speed is reduced. Since the residual architecture does not enhance the module variable, the complexity of the trained degradation and gradient disappearance is effectively mitigated, and the convergence efficacy of the module is improved. Thus, the SqueezeNet architecture was applied as a backbone network to extract features. Figure 2 showcases the framework of the SqueezeNet method. 

Compared with AlexNet and VGGNet, the SqueezeNet architecture has a smaller number of parameters. The fire module was the primary approach from SqueezeNet. This approach was classified into expand and squeeze structures. The squeeze encompasses 1×1 convolutional kernels. The expand layer includes 3×3 and 1×1 convolutional kernels. The number of 3×3 convolutional kernels is $E_{3\times 3}\;$ and the number of 1×1 convolutional kernels is $E_{1\times 1}.$ The model must satisfy $<\left(E_{1\times 1}+E_{3\times 3}\right)$. Thus, 1×1 convolution is added to each inception module, the number of input networks and the convolutional kernel variable are decreased, and the computation difficulty is reduced. Lastly, a 1×1 convolutional layer is added to enhance the number of channels and feature extraction. SqueezeNet changes 3×3 convolution with a 1×1 convolutional layer to reduce the variable count to one-ninth. Image feature extraction depends on a shared convolutional layer. The lowest-level features, such as edges and angles, are detached from the basic network. The higher-level features explain that the target form is eliminated at the highest level. For demonstrating the ship target on scale, the FPN was determined to extend the backbone network; viz., it was especially efficient in the detection of smaller targets. The topmost-level feature of FPN architecture is integrated with basic features by up-sampling via each layer predicting the feature map.

To adjust the hyperparameters of the SqueezeNet method, an AOA was implemented in this work. The AOA starts with a number of arbitrary populations of objects as candidates (immersed objects) [19]. Here, the object was initialized through arbitrary location from the fluid. The initial location of each object was accomplished as follows:

(1) $$x\left(i\right)=x_{l}\left(i\right)+rand\times \left(x_{u}\left(i\right)-x_{l}\left(i\right)\right)i=1,2,\;\ldots ,\;N$$

In this expression, $x\left(i\right)$ describes the $i^{th}$ object from a population with N objects, along with $x_{u}\left(i\right)$ and $x_{l}\left(i\right)$, which indicate the upper and lower boundaries of the solution space, respectively. In addition, the following indicates the location, AOA initialized density (*D*), acceleration (*A*), and volume (*V*), to *i^th^* object numbers:

(2) $$V\left(i\right)=rand\;$$

(3) $$D\left(i\right)=rand$$

(4) $$A\left(i\right)=x_{l}\left(i\right)+rand\times \left(x_{u}\left(i\right)-x_{l}\left(i\right)\right)\;$$

Next, the cost value of the candidate is evaluated and stored as $V^{best},$ $D^{best}$, or $A^{best}$, based on the population. Then, the candidate is upgraded through the parameter model as follows:

(5) $$V^{t+1}\left(i\right)=V^{t}\left(i\right)+rand\times \left(V^{best}-V^{t}\left(i\right)\right)$$

(6) $$D^{t+1}\left(i\right)=D^{t}\left(i\right)+rand\times \left(D^{best}-D^{t}\left(i\right)\right)$$

In this case, $y^{best}$ and $D^{best}$ denote the density and volume, respectively, associated with the best object initiated before, and rand indicates the arbitrary number that is uniformly distributed. The AOA applies a transfer operator (*TF*) to reach exploration–exploitation:

(7) $$TF=\;\exp\;\left(\frac{t-t^{\max}}{t^{\max}}\right)$$

In Equation (7), TF slowly steps up from the period still accomplishing 1, and t and $t^{max}$ indicate the iteration value and maximal iteration count, respectively. Likewise, a reduction factor of (*d*) density is used to offer a global–local search:

(8) $$D^{t+1}=\exp\left(\frac{t^{\max}-t}{t^{\max}}\right)-\left(\frac{t}{t^{max}}\right)$$

In Equation (8), $D^{t+1}$ is reduced with time that offers the ability to converge. This term renders a proper trade-off between exploitation and exploration. The exploration was stimulated on the basis of collision among objects. When TF≤0.5, a random material (mr) was preferred for upgrading acceleration of the object to t+1 iteration:

(9) $$A^{t+1}=\frac{D_{mr}+V_{mr}\times A_{mr}}{D^{t+1}\left(i\right)\times V^{t+1}\left(i\right)}$$

Here, $A\left(i\right)$, $V\left(i\right),$ and $D\left(i\right)$ denote the acceleration, volume, and density of the $i^{th}$ object. The exploitation was stimulated based on no collision among objects. When TF>0.5, the object is then upgraded as follows:

(10) $$A^{t+1}\left(i\right)=\frac{D^{best}+V^{best}\times A^{best}}{D^{t+1}\left(i\right)\times V^{t+1}\left(i\right)}$$

where $A^{best}$ indicates the optimal object acceleration. The subsequent step to normalize acceleration for assessing alteration percentage is as follows:

(11) $$A^{t+1}\left(\bar{i}\right)=u\times \frac{A^{t+1}\left(i\right)-\min\left(A\right)}{\max\left(A\right)-\min\left(A\right)}+l\;$$

Here, $A^{t+1}\left(i\right)$ refers to the percentage of steps, and l and u correspondingly imply the normalized limit that is fixed to 0.1 and 0. 9, respectively. When TF≤0.5, the location of the *i^th^* object to the succeeding round is accomplished as follows:

(12) $$x^{t+1}\left(i\right)=x^{t}\left(i\right)+c_{1}\times rand\times A^{t+1}\left(\bar{i}\right)\times D\times \left(x^{rand}-x^{t}\left(i\right)\right)$$

In Equation (12), $C_{1}$ denotes the constant corresponding to 2. In addition, when TF>0.5, the location of the object is upgraded:

(13) $$x^{t+1}\left(i\right)=x^{best^{t}}+F\times c_{2}\times rand\times A^{t+1}\left(\bar{i}\right)\times D\times \left(T\times x^{best}-x^{t}\left(i\right)\right)$$

In this expression, $c_{2}$ denotes a constant number corresponding to 6. T enhances with time from a range $\left[c_{3}\times 0.3,1\right]$ and obtains a determined percentage in the best location. This percentage slowly enhances to diminish the variance among optimum and present locations to offer an optimal balance between exploration and exploitation. F shows the flag for changing the motion path as

(14) $$F=\left\{\begin{array}{l} +1,\;if\;P\leq 0.5\\ +1,\;if\;P>0.5 \end{array}\right.$$

while

(15) $$P=2\times rand-c_{4}$$

Finally, the value of each object was assessed through a cost function and returned the optimal solution once the end state was satisfied.

The AOA method extracts a fitness function (FF) to receive enhanced classifier outcomes. It sets a positive value that signifies the superior outcome of the candidate’s solutions. In this work, the minimized classifier error rate is indicated as the FF, as provided in Equation (16).

(16) $$\begin{array}{c} fitness\left(x_{i}\right)=ClassifierErrorRate\left(x_{i}\right)\\ \;\;\;\;\;\;\;\;\;\;\;\;\;\;\;\;\;\;\;\;\;\;\;\;\;\;\;\;\;\;\;\;\;\;\;\;\;\;\;\;\;\;\;\;=\frac{number\;of\;misclassified\;samples}{Total\;number\;of\;samples}*100 \end{array}$$

### 3.2. Breast Cancer Classification Using Optimal DBN Model

Finally, an Adamax optimizer with the DBN method was applied for the breast cancer classification process (Algorithm 1). A DBN is a stack of RBM, excluding the primary RBM that has an undirected connection [20]. Significantly, this network architecture creates DL possibilities and reduces training complexity. The simple and effective layer-wise trained method was developed for DBN by Hinton. It consecutively trains layers and greedily trains by tying the weight of unlearned layers, applying CD to learn the weight of a single layer and iterating until all the layers are trained. Then, the network weight was finetuned through a two-pass up-down model, and this illustrates that the network learned without pretraining, since this phase implemented as regular and assisted with the supervised optimized problem. The energy constrained from the directed approach was calculated where the maximal energy was upper-bounded and accomplished equivalence, whether the network weight was tied or not, as follows:

(17) $$E\left(x^{0},\;h^{0}\right)=-\left(\;\log\;p\left(h^{0}\right)+\;\log\;p\left(x^{0}|h^{0}\right)\right)$$

(18) $$\log\;p\left(x^{0}\right)\geq \underset{\forall h^{0}}{\sum }Q(h^{0}|x^{0})(\;\log\;p\left(h^{0}\right)+\;\log\;p(x^{0}|h^{0}))-\underset{\forall h^{0}}{\sum }Q(h^{0}|x^{0})\;\log\;Q(h^{0}|x^{0})$$

(19) $$\frac{\partial \;\log\;p\left(x^{0}\right)}{\partial \xi_{n,m}}=\underset{\forall h^{0}}{\sum }Q\left(h^{0}|x^{0}\right)\log\;p\left(h^{0}\right)$$

Then, iteratively learning the weight of the network, the up-down approach was used to finetune the network weight. The wake-sleep approach is an unsupervised algorithm applied to train NNs from two phases: the “wake” phase was implemented on the feedforward path to compute weight and the “sleep” phase was executed on the feedback path. The up-down approach was executed to network for decreasing underfit that could usually be detected by a greedily trained network. Particularly in the primary phase, the weight on the directed connection was from named parameters or generative weight that can be adjusted by updating the weight utilizing CD, calculating the wake-phase probability, and sampling the states. Then, the prior layer was stochastically stimulated with top-down connections called inference weights or parameters. The sleep-stage probability was calculated, the state was sampled, and the result was estimated.

For optimizing the training efficacy of the DBN, the Adamax optimizer was employed for altering the hyperparameter values [21]:

(20) $$w_{t}^{i}=w_{t-1}^{i}-\frac{\eta }{v_{t}+\epsilon }\cdot {\hat{m}}_{t}$$

where

(21) $${\hat{m}}_{t}=\frac{m_{t}}{1-\beta_{1}^{t}}$$

(22) $$v_{t}=max\left(\beta_{2}\cdot v_{t-1},\left|G_{t}\right|\right)$$

(23) $$m_{t}=\beta_{1}m_{t-1}+\left(1-\beta_{1}\right)\;G\;$$

(24) $$G=\nabla_{w}C\left(w_{t}\right)$$

In this expression, η denotes the learning rate, $w_{t}$ represents the weight at t step, $C\left(.\right)$ indicates the cost function, and $\nabla_{w}C\left(w_{t}\right)$ specifies the gradient of the $w_{t}$ weight variable. $\beta_{i}$ is exploited to select the data needed for the old upgrade, where $\beta_{i}\in \left[0,1\right].$ $m_{t}$ and $v_{t}$ represent the first and second moments.
**Algorithm 1.** Pseudocode of Adamaxη: Rate of Learning $\beta_{1}$, $\beta_{2}\in$ [0,1): Exponential decomposing value to moment candidate $C\left(w\right)$: The cost function with variable w $w_{0}$: Primary parameter vector $m_{0}\leftarrow 0$ $u_{0}\leftarrow 0$ i←0 (Apply time step) while w does not converge apply i←i+1 $m_{i}\leftarrow \beta_{1}\cdot m_{i-1}+\left(1-\beta_{1}\right)\cdot \frac{\partial C}{\partial w}\left(w_{i}\right)$ $u_{i}\leftarrow \;\max\left(\beta_{2}\cdot u_{i-1},\;\left|\frac{\partial C}{\partial w}\left(w_{i}\right)\right|\right)$ $w_{i+1}\leftarrow w_{i}-\left(\eta /\left(1-\beta_{1}^{i}\right)\right)\cdot m_{i}/u_{i}$ end while displaying $w_{i}$ (end variable)

## 4. Experimental Validation

**Figure 3:**
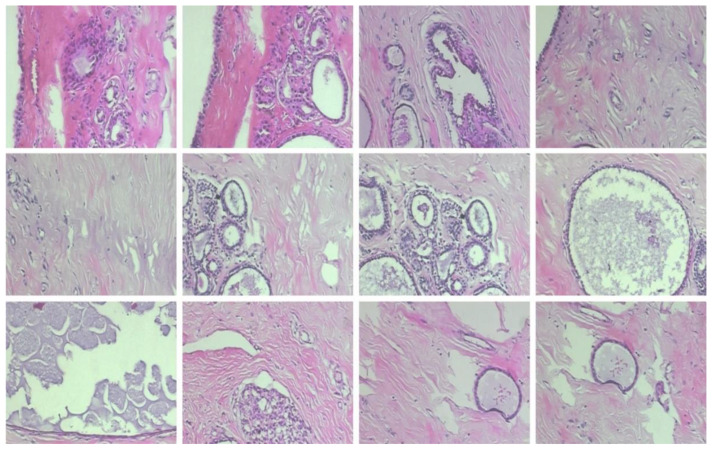
Sample images.

This section examines the breast cancer classification results of the AOADL-HBCC model on a benchmark dataset [22]. The dataset holds two sub-datasets, namely the 100× dataset and the 200× dataset, as represented in Table 1. Figure 3 illustrates some sample images.

The proposed model was simulated using Python 3.6.5 tools on PC i5-8600k, GeForce 1050Ti 4 GB, 16 GB RAM, 250 GB SSD, and 1 TB HDD. The parameter settings were given as follows: learning rate: 0.01, dropout: 0.5, batch size: 5, epoch count: 50, and activation: ReLU.

**Figure 4:**
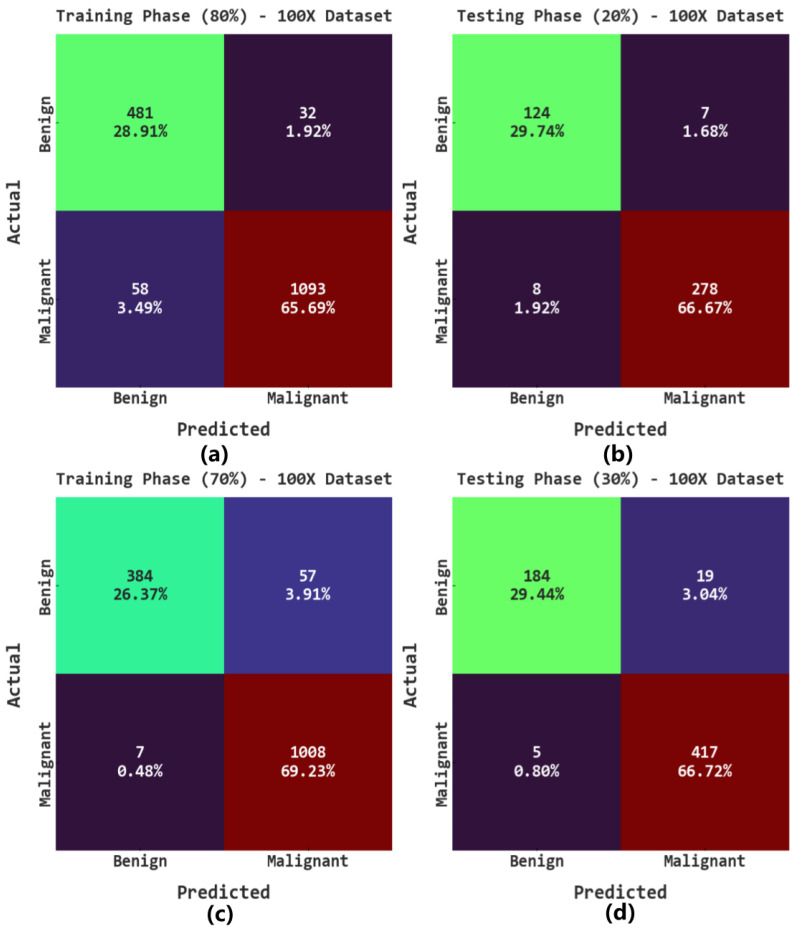
Confusion matrices of AOADL-HBCC system under 100× dataset: (**a**,**b**) TR and TS databases of 80:20, and (**c**,**d**) TR and TS databases of 70:30.

The confusion matrices of the AOADL-HBCC model on the 100× dataset are reported in Figure 4. This figure implies the AOADL-HBCC method proficiently recognized and sorted the HIs into malignant and benign classes in all aspects. 

Table 2 reports the overall breast cancer classification outcomes of the AOADL-HBCC method on the 100× database. The outcomes indicate that the AOADL-HBCC approach recognized both benign and malignant classes proficiently. For example, in the 80% TR database, the AOADL-HBCC method revealed an average $accu_{y}$ of 94.59%, $sens_{y}$ of 94.36%, $spec_{y}$ of 94.36%, $F_{score}$ of 93.75%, and MCC of 87.55%. Simultaneously, in the 20% TS database, the AOADL-HBCC method exhibited an average $accu_{y}$ of 96.40%, $sens_{y}$ of 95.93%, $spec_{y}$ of 95.93%, $F_{score}$ of 95.83%, and MCC of 91.67%. Concurrently, in the 70% TR database, the AOADL-HBCC approach displayed an average $accu_{y}$ of 95.60%, $sens_{y}$ of 93.19%, $spec_{y}$ of 93.19%, $F_{score}$ of 94.62%, and MCC of 89.56%.

**Figure 5:**
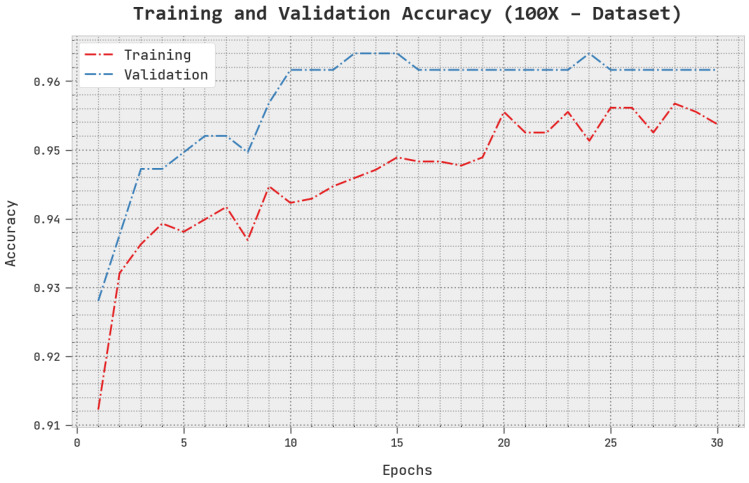
TACC and VACC analysis of AOADL-HBCC approach under 100× dataset.

The TACC and VACC of the AOADL-HBCC technique under the 100× dataset are inspected on BCC performance in Figure 5. This figure indicates that the AOADL-HBCC method displayed enhanced performance with increased values of TACC and VACC. It is noted that the AOADL-HBCC algorithm gained maximum TACC outcomes.

**Figure 6:**
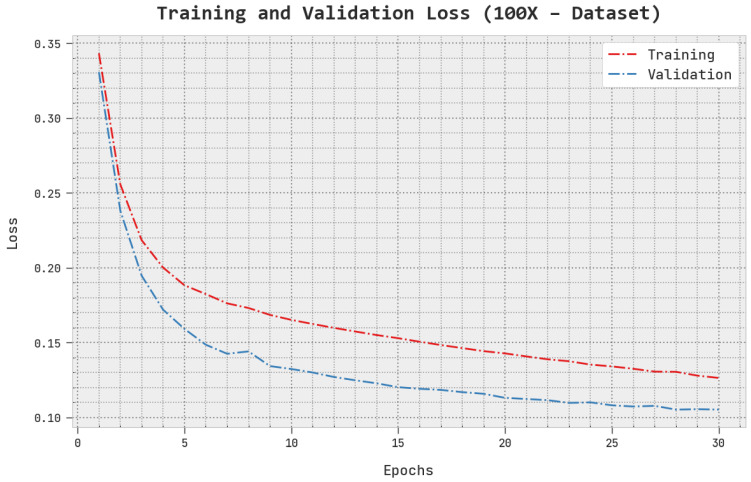
TLS and VLS analysis of AOADL-HBCC approach under 100× dataset.

The TLS and VLS of the AOADL-HBCC approach under the 100× dataset are tested on BCC performance in Figure 6. This figure shows that the AOADL-HBCC method exhibited better performance with minimal values of TLS and VLS. It is noted the AOADL-HBCC approach resulted in reduced VLS outcomes.

**Figure 7:**
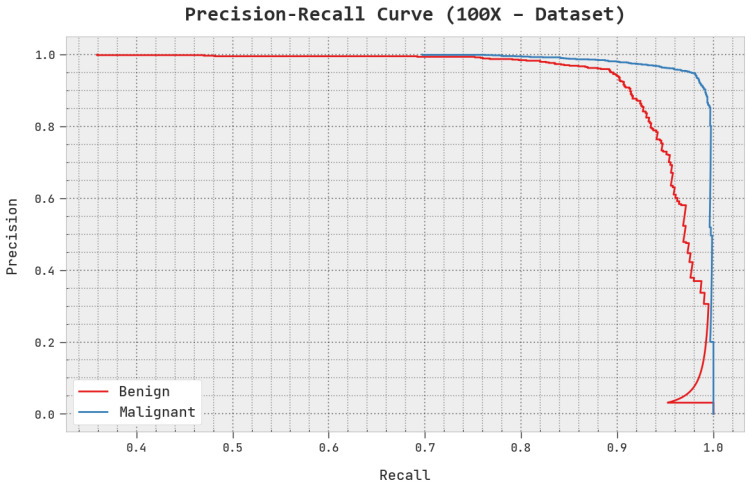
Precision–recall analysis of AOADL-HBCC approach under 100× dataset.

A clear precision–recall investigation of the AOADL-HBCC methodology under the test database is given in Figure 7. This figure exhibits that the AOADL-HBCC system enhanced precision–recall values in every class label.

**Figure 8:**
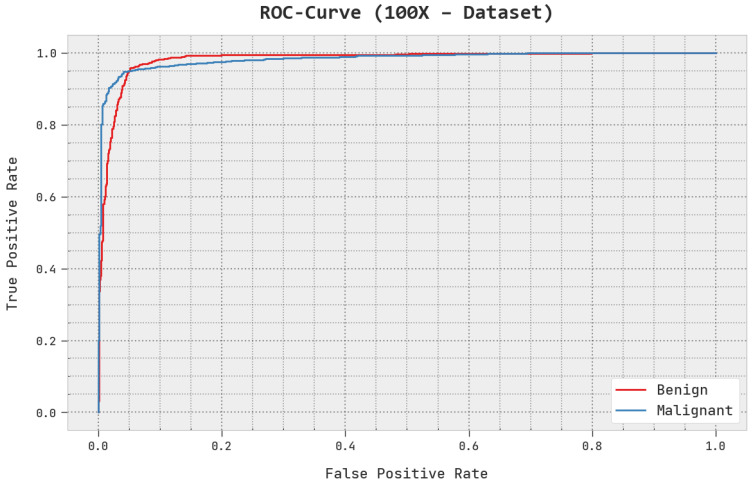
ROC analysis of AOADL-HBCC approach under 100× dataset.

A brief ROC analysis of the AOADL-HBCC approach under the test database is shown in Figure 8. The fallouts show that the AOADL-HBCC methodology exhibited its capacity in classifying different classes in the test database. 

**Figure 9:**
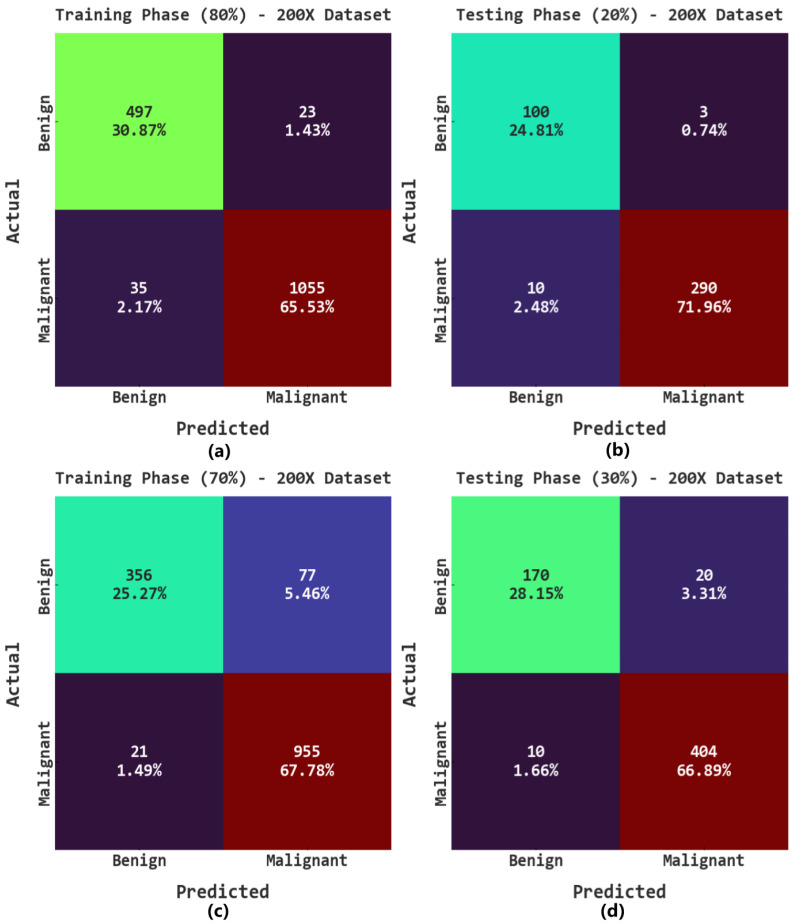
Confusion matrices of AOADL-HBCC system under 200× dataset: (**a**,**b**) TR and TS databases of 80:20, and (**c**,**d**) TR and TS databases of 70:30.

The confusion matrices of the AOADL-HBCC approach on the 200× database are given in Figure 9. This figure indicates that the AOADL-HBCC approach proficiently recognized and sorted the HIs into malignant and benign classes in every aspect. 

Table 3 shows the overall breast cancer classification results of the AOADL-HBCC approach on the 200× dataset. The results indicate that the AOADL-HBCC model recognized both benign and malignant classes proficiently. For example, in the 80% TR database, the AOADL-HBCC technique exhibited an average $accu_{y}$ of 96.40%, $sens_{y}$ of 96.18%, $spec_{y}$ of 96.18%, $F_{score}$ of 95.91%, and MCC of 91.83%. Concurrently, in the 20% TS database, the AOADL-HBCC approach displayed an average $accu_{y}$ of 96.77%, $sens_{y}$ of 96.88%, $spec_{y}$ of 96.88%, $F_{score}$ of 95.85%, and MCC of 91.80%. Simultaneously, in the 70% TR database, the AOADL-HBCC technique displayed an average $accu_{y}$ of 93.04%, $sens_{y}$ of 90.03%, $spec_{y}$ of 90.03%, $F_{score}$ of 91.51%, and MCC of 83.45%.

**Figure 10:**
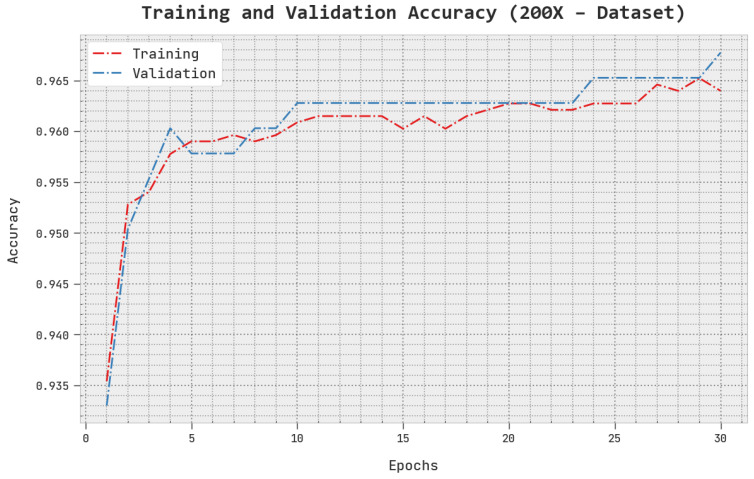
TACC and VACC analysis of AOADL-HBCC approach under 200× dataset.

The TACC and VACC of the AOADL-HBCC method under the 200× dataset are inspected on BCC performance in Figure 10. This figure shows that the AOADL-HBCC methodology displayed enhanced performance with increased values of TACC and VACC. It is noted that the AOADL-HBCC technique attained maximum TACC outcomes.

**Figure 11:**
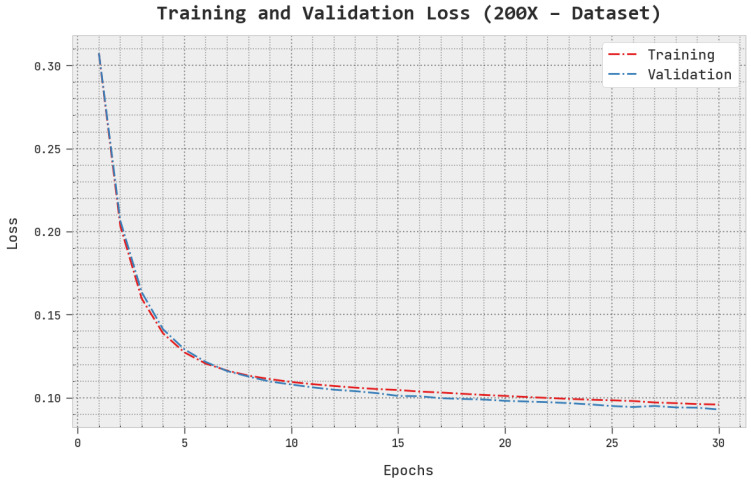
TLS and VLS analysis of AOADL-HBCC approach under 200× dataset.

The TLS and VLS of the AOADL-HBCC approach under the 200× dataset are tested on BCC performance in Figure 11. This figure indicates that the AOADL-HBCC methodology revealed superior performance with minimal values of TLS and VLS. It is noted that the AOADL-HBCC method resulted in reduced VLS outcomes.

**Figure 12:**
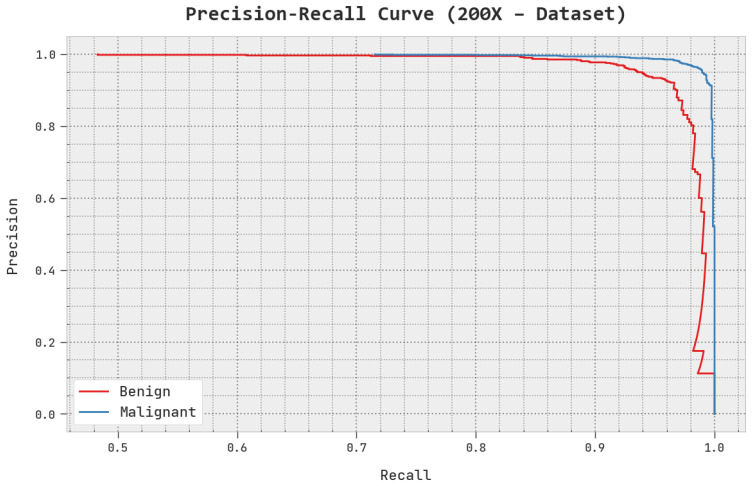
Precision–recall analysis of AOADL-HBCC approach under 200× dataset.

A clear precision–recall inspection of the AOADL-HBCC methodology under the test database is shown in Figure 12. This figure indicates that the AOADL-HBCC method enhanced precision–recall values in every class label.

**Figure 13:**
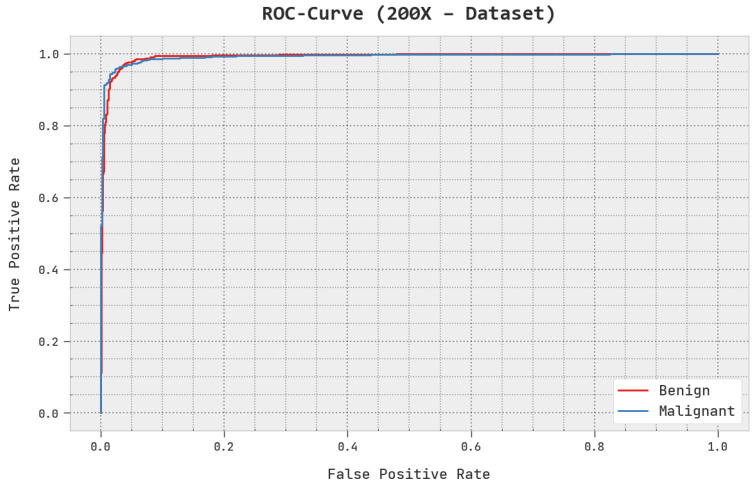
ROC analysis of AOADL-HBCC approach under 200× dataset.

A brief ROC study of the AOADL-HBCC system under the test database is given in Figure 13. The outcomes exhibited by the AOADL-HBCC method reveal its ability in classifying different classes in the test database. 

**Figure 14:**
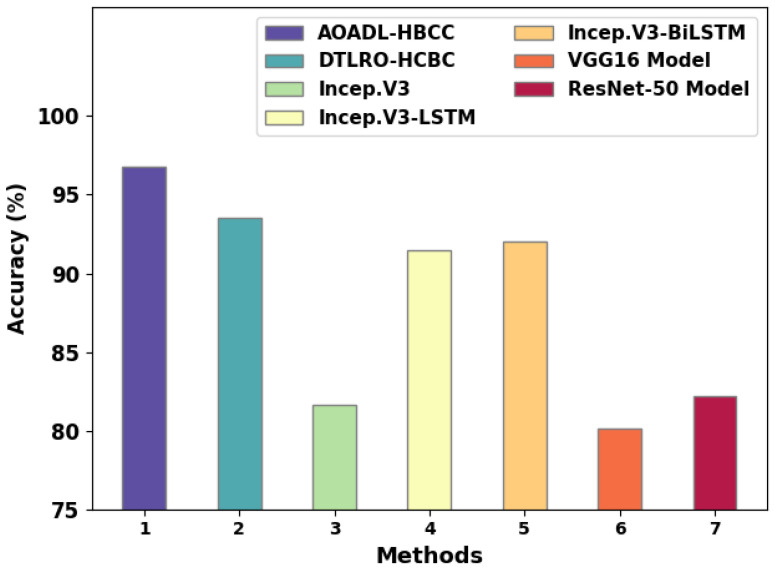
Comparative analysis of AOADL-HBCC system with existing approaches.

A detailed comparative study of the AOADL-HBCC model with recent DL models is reported in Table 4 and Figure 14 [23]. The simulation values representing the Incep. V3, VGG16, and ResNet-50 models reported lower $accu_{y}$ of 81.67%, 80.15%, and 82.18%, respectively. Next, the Incep. V3-LSTM and Incep. V3-BiLSTM models attained reasonable $accu_{y}$ of 91.46% and 92.05%, respectively. 

Although the DTLRO-HCBC model reached near-optimal $accu_{y}$ of 93.52%, the AOADL-HBCC model gained maximum $accu_{y}$ of 96.77%. These results ensured the enhanced outcomes of the AOADL-HBCC model over other models.

## 5. Conclusions

In this work, an automated breast cancer classification model, named the AOADL-HBCC technique, was developed on HIs. The presented AOADL-HBCC technique mainly aims to recognize the presence of breast cancer in HIs. At the primary level, the AOADL-HBCC technique exploited MF-based noise removal and a contrast enhancement process. In addition, the presented AOADL-HBCC technique utilized an AOA with a SqueezeNet model to derive feature vectors. Lastly, an Adamax optimizer with a DBN model was applied for the breast cancer classification process. In order to exhibit the enhanced breast cancer classification results of the AOADL-HBCC methodology, a wide range of simulations were performed. A comparative study indicated the better performance of the AOADL-HBCC technique over other recent methodologies, with a maximum accuracy of 96.77%. Therefore, the AOADL-HBCC technique can be employed for timely and accurate BC classification. In the future, ensemble-learning-based DL classifiers can be involved to boost the overall performance of the AOADL-HBCC technique. In addition, the performance of the proposed model can be tested on large-scale real-time databases.

## Figures and Tables

**Table 1:** Dataset details.

Class	No. of Images	
	100×	200×
Benign	644	623
Malignant	1437	1390
Total No. of Images	2081	2013

**Table 2:** BCC outcomes of AOADL-HBCC approach with various measures under 100× dataset.

Class	Accuracy	Sensitivity	Specificity	F-Score	MCC
Training/Testing (80:20)					
Training Phase					
Benign	94.59	93.76	94.96	91.44	87.55
Malignant	94.59	94.96	93.76	96.05	87.55
Average	94.59	94.36	94.36	93.75	87.55
Testing Phase					
Benign	96.40	94.66	97.20	94.30	91.67
Malignant	96.40	97.20	94.66	97.37	91.67
Average	96.40	95.93	95.93	95.83	91.67
Training/Testing (70:30)					
Training Phase					
Benign	95.60	87.07	99.31	92.31	89.56
Malignant	95.60	99.31	87.07	96.92	89.56
Average	95.60	93.19	93.19	94.62	89.56
Testing Phase					
Benign	96.16	90.64	98.82	93.88	91.21
Malignant	96.16	98.82	90.64	97.20	91.21
Average	96.16	94.73	94.73	95.54	91.21

**Table 3:** BCC outcomes of AOADL-HBCC approach with various measures under 200× dataset.

Class	Accuracy	Sensitivity	Specificity	F-Score	MCC
Training/Testing (80:20)					
Training Phase					
Benign	96.40	95.58	96.79	94.49	91.83
Malignant	96.40	96.79	95.58	97.32	91.83
Average	96.40	96.18	96.18	95.91	91.83
Testing Phase					
Benign	96.77	97.09	96.67	93.90	91.80
Malignant	96.77	96.67	97.09	97.81	91.80
Average	96.77	96.88	96.88	95.85	91.80
Training/Testing (70:30)					
Training Phase					
Benign	93.04	82.22	97.85	87.90	83.45
Malignant	93.04	97.85	82.22	95.12	83.45
Average	93.04	90.03	90.03	91.51	83.45
Testing Phase					
Benign	95.03	89.47	97.58	91.89	88.38
Malignant	95.03	97.58	89.47	96.42	88.38
Average	95.03	93.53	93.53	94.16	88.38

**Table 4:** Comparative analysis of AOADL-HBCC system with current approaches.

Methods	Accuracy
AOADL-HBCC	96.77
DTLRO-HCBC	93.52
Incep.V3	81.67
Incep.V3-LSTM	91.46
Incep.V3-BiLSTM	92.05
VGG16 Model	80.15
ResNet-50 Model	82.18

## Data Availability

Data sharing is not applicable to this article as no datasets were generated during the current study.

## References

[1] Boumaraf S., Liu X., Zheng Z., Ma X., Ferkous C. (2021). A new transfer learning based approach to magnification dependent and independent classification of breast cancer in histopathological images. Biomed. Signal Process. Control.

[2] Bose S., Garg A., Singh S.P. Transfer Learning for Classification of Histopathology Images of Invasive Ductal Carcinoma in Breast. Proceedings of the 2022 3rd International Conference on Electronics and Sustainable Communication Systems (ICESC).

[3] Ahmad N., Asghar S., Gillani S.A. (2021). Transfer learning-assisted multi-resolution breast cancer histopathological images classification. Vis. Comput..

[4] Thuy M.B.H., Hoang V.T. (2019). Fusing of deep learning, transfer learning and gan for breast cancer histopathological image classification. International Conference on Computer Science, Applied Mathematics and Applications.

[5] Abbasniya M.R., Sheikholeslamzadeh S.A., Nasiri H., Emami S. (2022). Classification of Breast Tumors Based on Histopathology Images Using Deep Features and Ensemble of Gradient Boosting Methods. Comput. Electr. Eng..

[6] Chang J., Yu J., Han T., Chang H.J., Park E. A method for classifying medical images using transfer learning: A pilot study on histopathology of breast cancer. Proceedings of the 2017 IEEE 19th International Conference on E-Health Networking, Applications and Services (Healthcom).

[7] Ahmad H.M., Ghuffar S., Khurshid K. Classification of breast cancer histology images using transfer learning. Proceedings of the 2019 16th International Bhurban Conference on Applied Sciences and Technology (IBCAST).

[8] Alzubaidi L., Al-Shamma O., Fadhel M.A., Farhan L., Zhang J., Duan Y. (2020). Optimizing the performance of breast cancer classification by employing the same domain transfer learning from hybrid deep convolutional neural network model. Electronics.

[9] Baghdadi N.A., Malki A., Balaha H.M., AbdulAzeem Y., Badawy M., Elhosseini M. (2022). Classification of breast cancer using a manta-ray foraging optimized transfer learning framework. PeerJ Comput. Sci..

[10] Sajjad U., Hussain I., Hamid K., Ali H.M., Wang C.C., Yan W.M. (2022). Liquid-to-vapor phase change heat transfer evaluation and parameter sensitivity analysis of nanoporous surface coatings. Int. J. Heat Mass Transf..

[11] Shankar K., Dutta A.K., Kumar S., Joshi G.P., Doo I.C. (2022). Chaotic Sparrow Search Algorithm with Deep Transfer Learning Enabled Breast Cancer Classification on Histopathological Images. Cancers.

[12] Deniz E., Şengür A., Kadiroğlu Z., Guo Y., Bajaj V., Budak Ü. (2018). Transfer learning based histopathologic image classification for breast cancer detection. Health Inf. Sci. Syst..

[13] Khan S., Islam N., Jan Z., Din I.U., Rodrigues J.J.C. (2019). A novel deep learning based framework for the detection and classification of breast cancer using transfer learning. Pattern Recognit. Lett..

[14] Talo M. (2019). Automated classification of histopathology images using transfer learning. Artif. Intell. Med..

[15] Singh R., Ahmed T., Kumar A., Singh A.K., Pandey A.K., Singh S.K. (2020). Imbalanced breast cancer classification using transfer learning. IEEE/ACM Trans. Comput. Biol. Bioinform..

[16] Fan J., Lee J., Lee Y. (2021). A transfer learning architecture based on a support vector machine for histopathology image classification. Appl. Sci..

[17] Elmannai H., Hamdi M., AlGarni A. (2021). Deep learning models combining for breast cancer histopathology image classification. Int. J. Comput. Intell. Syst..

[18] Escorcia-Gutierrez J., Gamarra M., Beleño K., Soto C., Mansour R.F. (2022). Intelligent deep learning-enabled autonomous small ship detection and classification model. Comput. Electr. Eng..

[19] Kaveh A., Hamedani K.B. (2022). Improved arithmetic optimization algorithm and its application to discrete structural optimization. Structures.

[20] Zand R., Camsari K.Y., Pyle S.D., Ahmed I., Kim C.H., DeMara R.F. Low-energy deep belief networks using intrinsic sigmoidal spintronic-based probabilistic neurons. Proceedings of the 2018 on Great Lakes Symposium on VLSI.

[21] Kandel I., Castelli M., Popovič A. (2020). Comparative study of first order optimizers for image classification using convolutional neural networks on histopathology images. J. Imaging.

[22] https://web.inf.ufpr.br/vri/databases/breast-cancer-histopathological-database-breakhis/.

[23] Ragab M., Nahhas A.F. (2022). Optimal Deep Transfer Learning Model for Histopathological Breast Cancer Classification. CMC-Comput. Mater. Contin..

